# The role of mesenchymal stem cells in cancer and prospects for their use in cancer therapeutics

**DOI:** 10.1002/mco2.663

**Published:** 2024-07-28

**Authors:** Jian Tang, Yu Chen, Chunhua Wang, Ying Xia, Tingyu Yu, Mengjun Tang, Kun Meng, Lijuan Yin, Yang Yang, Liang Shen, Hui Xing, Xiaogang Mao

**Affiliations:** ^1^ Central Laboratory Xiangyang Central Hospital Affiliated Hospital of Hubei University of Arts and Science Xiangyang China; ^2^ Medical Affairs, Xiangyang Central Hospital Affiliated Hospital of Hubei University of Arts and Science Xiangyang China; ^3^ Department of Clinical Laboratory Xiangyang No. 1 People's Hospital Hubei University of Medicine Xiangyang Hubei Province China; ^4^ State Key Laboratory of Food Nutrition and Safety Key Laboratory of Industrial Microbiology Ministry of Education Tianjin Key Laboratory of Industry Microbiology National and Local United Engineering Lab of Metabolic Control Fermentation Technology China International Science and Technology Cooperation Base of Food Nutrition/Safety and Medicinal Chemistry College of Biotechnology Tianjin University of Science & Technology Tianjin China; ^5^ Shenzhen Key Laboratory of Pathogen and Immunity National Clinical Research Center for Infectious Disease State Key Discipline of Infectious Disease Shenzhen Third People's Hospital Second Hospital Affiliated to Southern University of Science and Technology Shenzhen China; ^6^ Department of Obstetrics and Gynecology Xiangyang Central Hospital Affiliated Hospital of Hubei University of Arts and Sciences Xiangyang China

**Keywords:** drug delivery, mesenchymal stem cells, tumor microenvironment, tumor‐targeted therapy

## Abstract

Mesenchymal stem cells (MSCs) are recruited by malignant tumor cells to the tumor microenvironment (TME) and play a crucial role in the initiation and progression of malignant tumors. This role encompasses immune evasion, promotion of angiogenesis, stimulation of cancer cell proliferation, correlation with cancer stem cells, multilineage differentiation within the TME, and development of treatment resistance. Simultaneously, extensive research is exploring the homing effect of MSCs and MSC‐derived extracellular vesicles (MSCs‐EVs) in tumors, aiming to design them as carriers for antitumor substances. These substances are targeted to deliver antitumor drugs to enhance drug efficacy while reducing drug toxicity. This paper provides a review of the supportive role of MSCs in tumor progression and the associated molecular mechanisms. Additionally, we summarize the latest therapeutic strategies involving engineered MSCs and MSCs‐EVs in cancer treatment, including their utilization as carriers for gene therapeutic agents, chemotherapeutics, and oncolytic viruses. We also discuss the distribution and clearance of MSCs and MSCs‐EVs upon entry into the body to elucidate the potential of targeted therapies based on MSCs and MSCs‐EVs in cancer treatment, along with the challenges they face.

## Graphical abstract

Malignant tumor cells regulate pathways such as immune response, angiogenesis, and differentiation of cancer‐associated fibroblasts within the tumor microenvironment by recruiting mesenchymal stem cells (MSCs), thereby promoting their own survival and further progression. Leveraging the tumor homing properties of MSCs and the extracellular vesicles (EVs) they secrete, engineered MSCs and EVs are being developed to deliver antitumor substances for precise targeted anticancer therapy.

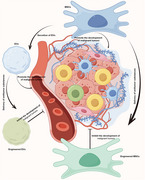

## 1 INTRODUCTION

Noncommunicable diseases stand as the predominant contributors to global mortality, with cancer or malignant tumor emerging as a foremost contender among them.^1^, ^2^, ^3^ The landscape of cancer treatment has undergone significant transformation since the onset of the 21st century, yielding considerable enhancements in patient outcomes.^4^ Notwithstanding these recent therapeutic strides in cancer management, metastasis continues to account for over 90% of cancer‐related fatalities.^5^ The progression of cancer is closely associated with the tumor microenvironment (TME), immune evasion, epithelial–mesenchymal transition (EMT), and tumor drug resistance.^6^, ^7^, ^8^, ^9^, ^10^, ^11^, ^12^ Therefore, there is an urgent need to find new targeted strategies for more effective cancer treatment.

Numerous studies have indicated the potential involvement of mesenchymal stem cells (MSCs) in the progression of tumors.^13^, ^14^ MSCs, recognized as a promising reservoir of multipotent stem cells endowed with high self‐renewal and differentiation capabilities, emerge as a hopeful candidate for cell therapy‐based regenerative medicine.^15^, ^16^ The therapeutic characteristics of MSCs are closely related to their potential for trans‐differentiation, secretion of trophic factors, and immune regulation.^17^, ^18^, ^19^, ^20^ They have been widely used in the treatment of degenerative diseases such as degenerative disc disease, Alzheimer's disease, multiple sclerosis, Parkinson's disease, type I diabetes and myocardial infarction.^21^, ^22^, ^23^, ^24^, ^25^, ^26^, ^27^, ^28^, ^29^, ^30^, ^31^, ^32^ However, as a key component of the TME, MSCs are multipotent stromal stem cells, play a crucial role in influencing TME formation and tumor progression in most cancers.^33^, ^34^, ^35^ MSCs can be recruited to the TME through tumor‐homing, gaining the ability to dynamically influence cancer progression.^36^, ^37^, ^38^, ^39^ Tumor growth can be supported by them, cancer cell migration facilitated, immune responses suppressed, angiogenesis promoted, differentiation into tumor‐associated fibroblasts facilitated, EMT promoted, interaction with cancer stem cells (CSC) facilitated, and drug resistance induced in cancer.^14^, ^40^, ^41^

Chemotherapy is currently one of the effective and primary methods for antitumor treatment. However, the systemic toxic side effects resulting from poor drug targeting limit the application of chemotherapy drugs. Therefore, numerous researchers are focusing on exploring precise targeted drug delivery systems for tumors to enhance antitumor effects and reduce drug toxic side effects.^42^, ^43^, ^44^ Interestingly, due to the homing ability of MSCs toward tumors, an increasing number of studies have found that this tumor homing capability of MSCs is highly suitable for targeted delivery of antitumor drugs to tumor tissues.^45^ Additionally, extracellular vesicles derived from MSCs (MSCs‐EVs) also possess tumor homing capabilities similar to MSCs, and researchers have conducted a series of studies using MSCs‐EVs for delivering antitumor substances.^46^ It is evident that MSCs and MSCs‐EVs possess significant potential in the precise targeted delivery of antitumor substances.

In this review, we first outline the role and potential mechanisms of tumor‐recruited MSCs in the latest research, as well as the multifaceted impact of MSCs within the TME (including immune response, angiogenesis, cancer cell proliferation, correlation with CSCs, multilineage differentiation within the TME, and treatment resistance) in promoting malignant tumor development and the associated molecular mechanisms. Additionally, we discuss the prospects and challenges of using MSCs and MSCs‐EVs as carriers for antitumor substances in cancer therapy, and analyze the distribution and clearance of MSCs and MSCs‐EVs after administration to clarify their feasibility, safety, and challenges.

## 2 MSCS IN THE TME

### 2.1 Source and characteristics of MSCs

Stem cells are a class of undifferentiated cells with self‐renewal capacity and multidifferentiation potential, which are relatively primitive cells that can differentiate into cells with diverse functions under specific conditions.^47^, ^48^ According to their differentiation potential, stem cells can be divided into totipotent, pluripotent, and multipotent stem cells.^49^ Based on the developmental stage, stem cells can be classified into embryonic stem cells and adult stem cells (somatic stem cells).^50^ MSCs are a type of adult multipotent stem cells originating from the mesoderm.^51^, ^52^

MSCs were initially discovered in the bone marrow^53^, ^54^ and have been later confirmed in other organs or tissues, such as adipose tissue, liver, myocardium, lungs, adrenal glands, and pancreas, among others.^55^, ^56^ Even with extensive research over the years, there is still no comprehensive understanding of how to classify bone marrow MSCs in vitro. Morphological observations of cultured cells show a diversity of cell populations, consisting of fibroblast‐like cells, round cells, and flattened cells.^57^ According to the minimal guidelines for defining human MSC identity published by the International Society for Cellular Therapy, the isolated cells are typically positive (over 95%) for CD73, CD90, and CD105, while being negative (less than 2%) for CD14, CD19, CD34, CD45, CD79α, CD11b, and HLA‐DR.^58^, ^59^ Furthermore, these cells have the ability to differentiate into specific lineages, such as osteoblasts, adipocytes, or chondrocytes, and display plasticity during in vitro culture.^60^, ^61^, ^62^

As a consequence of the recruitment effect imposed by tumor cells on MSCs, TME is characterized by an abundant MSC population. It has been proposed that MSCs may constitute 0.01–5% of the TME population.^63^ Current research suggests that MSCs present in tumor stroma originate from both local and distant sources.^64^, ^65^ During the early stages of tumor formation, tissue resident MSCs are initially recruited to the TME and reprogrammed into cancer‐associated MSCs (CA‐MSCs) that facilitate tumor growth.^66^, ^67^ Given that MSCs reside in numerous tissues throughout the body and are in closer proximity to the primary tumor site, these MSC populations are most likely to represent the initial cohort inhabiting the TME and supporting early tumor development as CA‐MSCs.^66^

As tumor progresses, the levels of inflammation and hypoxia in TME increase, leading to the potential recruitment of MSCs from other tissues to further facilitate tumor development.^68^, ^69^ Although the impact of CA‐MSCs on both tumors and TME has been well documented, the specific origins of MSCs recruited by tumor cells remain unclear. Current evidence suggests that bone marrow may serve as a crucial tissue source of MSCs recruited by tumor cells.^70^ Karnoub et al.^71^ demonstrated the ability of breast cancer cells to attract bone marrow‐derived MSCs (BM‐MSCs) and promote their progression. However, MSCs from other tissues also have the potential to be recruited by tumor cells. For example, adipose tissue‐derived MSCs (AD‐MSCs) from breast fat tissue can also be recruited by breast cancer cells to the TME, significantly promoting breast cancer development.^72^

Given the abundant presence of MSCs in various body tissues, the tissue‐resident MSCs identified within the TME are likely the initial population to colonize the TME and stimulate cancer cell proliferation.^73^, ^74^, ^75^ As the tumor progresses, MSCs from other tissues are also attracted to the TME, further promoting tumor development. In the subsequent sections, we will delve into the mechanisms underlying the homing of MSCs to tumor tissues.

### 2.2 MSC homing to cancer tissue

MSCs play a crucial role in tissue reparation, inflammation, proliferation, and remodeling of tissue are all stages of regeneration involving MSCs.^76^, ^77^, ^78^, ^79^, ^80^ On the other hand, tumors are sometimes described as “non‐healing wounds” that require host to “heal” them.^81^ The process of wound healing is characterized by hemostasis to control bleeding, inflammation (both humoral and cellular) to cleanse the wound, angiogenesis for the formation of new blood vessels, and the generation of mature connective tissue stroma.^82^ Remarkably, as a result of their homing capacity, MSCs are able to migrate to areas of injury and inflammation and undergo differentiation, promoting structural and functional repair.^83^, ^84^

**FIGURE 1:**
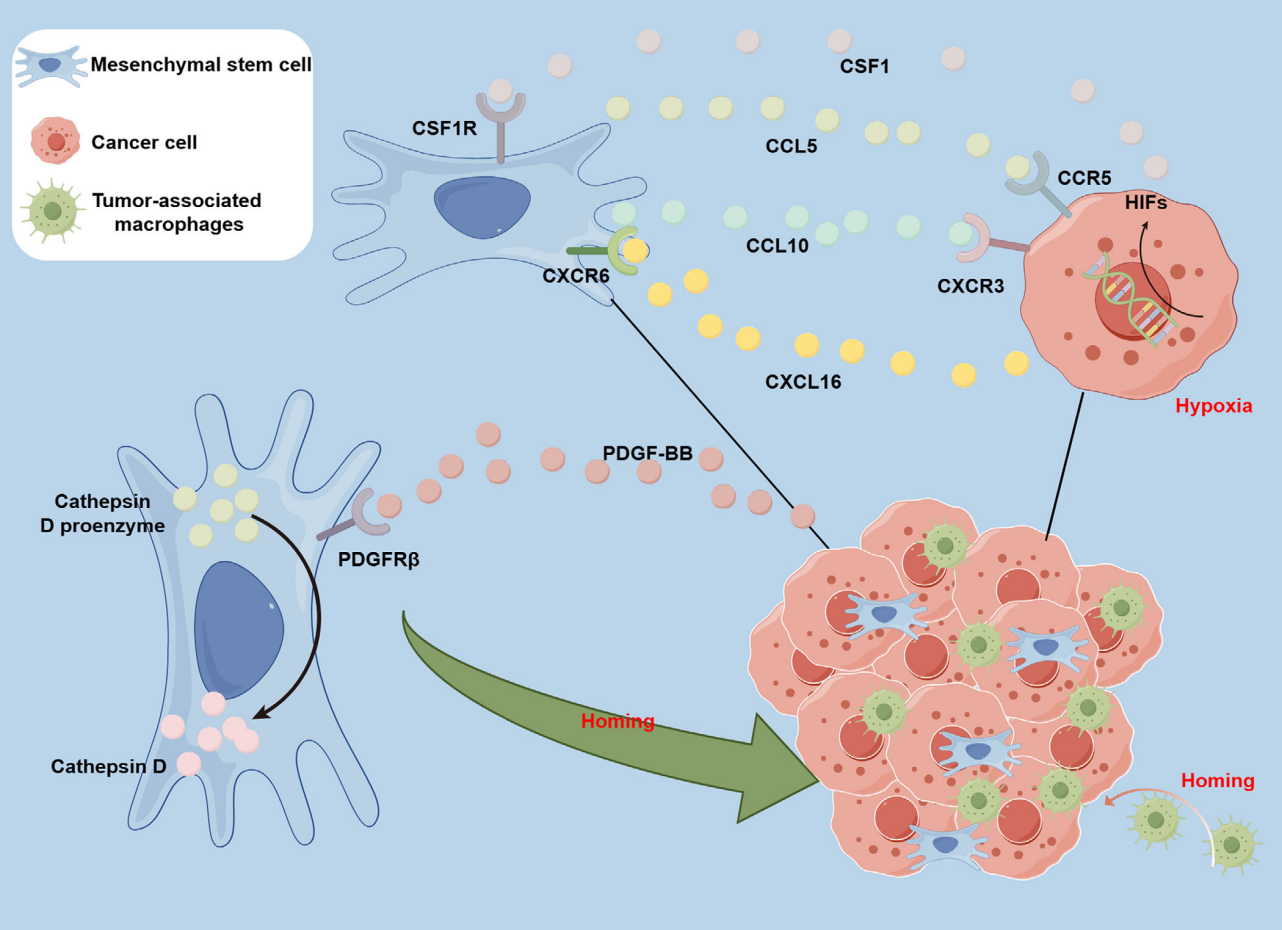
Molecular mechanism of MSCs homing into malignant tumor. In malignant tumor, the activation of Cathepsin D proenzyme within the MSCs is induced, leading them to home toward malignant tumor cells; Secretion of PDGF‐BB by malignant tumor cells facilitates the binding of PDGFRβ on the surface of MSCs, promoting their attraction to malignant tumor sites; hypoxia in the malignant tumor microenvironment results in increased expression of HIFs, which in turn induces malignant tumor cells to secrete CXCL16. This chemokine binds to CXCR6 on the surface of MSCs and triggers their secretion of CXCL10. The interaction between CXCL10 and CXCR3 on malignant tumor cells further drives the homing of MSCs toward malignant tumor cells. Subsequently, MSCs secrete CCL5, which binds to CCR5 on malignant tumor cells, inducing the secretion of CSF1. The binding of CSF1 to the CSF1 receptor on the membrane of MSCs promotes the recruitment of tumor‐associated macrophages and myeloid‐derived suppressor cells to the TME.

Recent studies have revealed that the homing of MSCs to cancer cells is a highly intricate process, yielding fascinating discoveries (Figure 1). It has been demonstrated that MSCs exhibit tropism toward various types of tumors, including but not limited to hepatocellular carcinoma, lung cancer, breast cancer, cervical cancer, pancreatic cancer, prostate cancer, glioblastoma, Ewing sarcoma, osteosarcoma, and melanoma.^71^, ^85^, ^86^, ^87^, ^88^, ^89^, ^90^, ^91^, ^92^, ^93^, ^94^ The specific mechanisms underlying the homing of MSCs to tumor tissues remain to be fully elucidated. It is generally believed that the homing process of MSCs is initiated by the release of various cells and chemoattractant factors by tumor tissues, which activate relevant receptors on MSCs, thereby recruiting MSCs to migrate toward tumor tissues.^95^, ^96^ Among these, the study of chemoattractant factors involved in MSC tumor homing has been particularly insightful.

Research suggests that the interaction between chemoattractant factors released by tumor tissues and their corresponding receptors on MSCs is a critical determinant in inducing the targeted migration of MSCs toward tumor tissues.^97^ According to Camorani et al.’s^98^ research, breast cancer cells release platelet‐derived growth factor BB (PDGFBB), which interacts with the platelet‐derived growth factor receptor β (PDGFRβ) on the surface of BM‐MSCs to facilitate the cells' homing to breast cancer cells. However, when BM‐MSCs were treated with a nuclease‐resistant RNA aptamer targeting PDGFRβ, the signaling pathways dependent on this receptor were inhibited, leading to a significant impairment in the homing of BM‐MSCs to breast cancer cells.^98^ These findings suggest that the anti‐PDGFRβ aptamer represents a novel therapeutic tool that can disrupt the recruitment ability of triple‐negative breast cancer cells to BM‐MSCs, thereby providing a theoretical basis for further exploration of aptamers in more complex preclinical environments.

Moreover, several reports suggested that the homing of MSCs to cancer tissue is associated with hypoxia in the TME. Tumor oxygenation is both variable and significantly reduced when compared with normal tissue. This is caused by the rapid proliferation of tumor cells, leading to hypoxia due to insufficient perfusion and diffusion within the TME.^99^, ^100^, ^101^ Egea et al.’s^102^ research has unveiled the potential molecular mechanisms by which hypoxia induces the migration of MSCs toward tumor cells. Their work discovered that hypoxia increases the expression of hypoxia‐inducible factor 1 alpha (HIF‐1α) in MSCs, thereby enhancing their ability to migrate toward tumor cells.^102^ Similarly, the development of tumors led to elevated expression of HIFs within the TME, attributed to increased hypoxia.^103^, ^104^, ^105^, ^106^ In summary, the process of malignant tumor cell invasion and metastasis involves several key steps. Initially, hypoxia causes the secretion of CXCL16 by cancer cells, which subsequently binds to the membrane receptor CXCR6 on MSCs. This binding initiates the secretion of CXCL10 by MSCs, which, in turn, drives the recruitment of MSCs to cancer cells via the receptor CXCR3 on the cell membrane. Subsequently, homing MSCs secrete CCL5, which binds to CCR5 on cancer cells, inducing the secretion of CSF1 by cancer cells. CSF1 then binds to the CSF1 receptor on the membrane of MSCs, ultimately leading to the recruitment of tumor‐associated macrophages and myeloid‐derived suppressor cells, thereby promoting cancer cell invasion and metastasis.^107^, ^108^

In brief, MSCs are recruited to the TME of cancer through the various pathways as mentioned above, thereby augmenting the population of MSCs in that vicinity. These alterations ultimately contribute to the progressive deterioration of cancer.

## 3 MSCs IN CANCER DEVELOPMENT

The characterization of tumor properties is determined by the heterogeneity of the TME.^109^, ^110^, ^111^ The TME is an intricate network consisting of cells and molecules that undergo cellular and physical alterations within the host tissue to facilitate the growth of tumors and the progression of cancer.^112^, ^113^, ^114^, ^115^ The TME encompasses the non‐neoplastic compartment surrounding tumor tissues. The specific composition of the TME varies depending on the type of tumors under consideration, but it notably includes immune cells, pericytes, vascular smooth muscle cells, endothelial cells, myofibroblasts, occasionally adipocytes, fibroblasts, and MSCs.^116^, ^117^, ^118^, ^119^ TEM engage in intercellular communication via the secretion of mediators and the production of extracellular matrix (ECM), thereby facilitating the dynamic ecosystem necessary for cancer progression.^120^, ^121^ Tumor parenchyma cells grow symbiotically by interacting with the surrounding stroma through paracrine and juxtacrine signaling events.^122^, ^123^, ^124^, ^125^ Interestingly, MSCs play a significant role in regulating the TME.^126^ And the production of these effects by MSCs may be through the release of extracellular vehicles (EVs) and diverse cytokines.^127^, ^128^

The intricate interplay between malignant tumor cells and various resident cells leads to a highly complex intercellular communication network within the TME. The EVs and cytokines released by MSCs are widely recognized as the principal mediators that facilitate cell‐to‐cell communication in the TME.^40^, ^129^ As a result, MSCs are recruited by cancer cells into the TME and may exert a significant impact on the progression of cancer through the secretion of EVs and cytokines.

### 3.1 Suppression of the immune response

**FIGURE 2:**
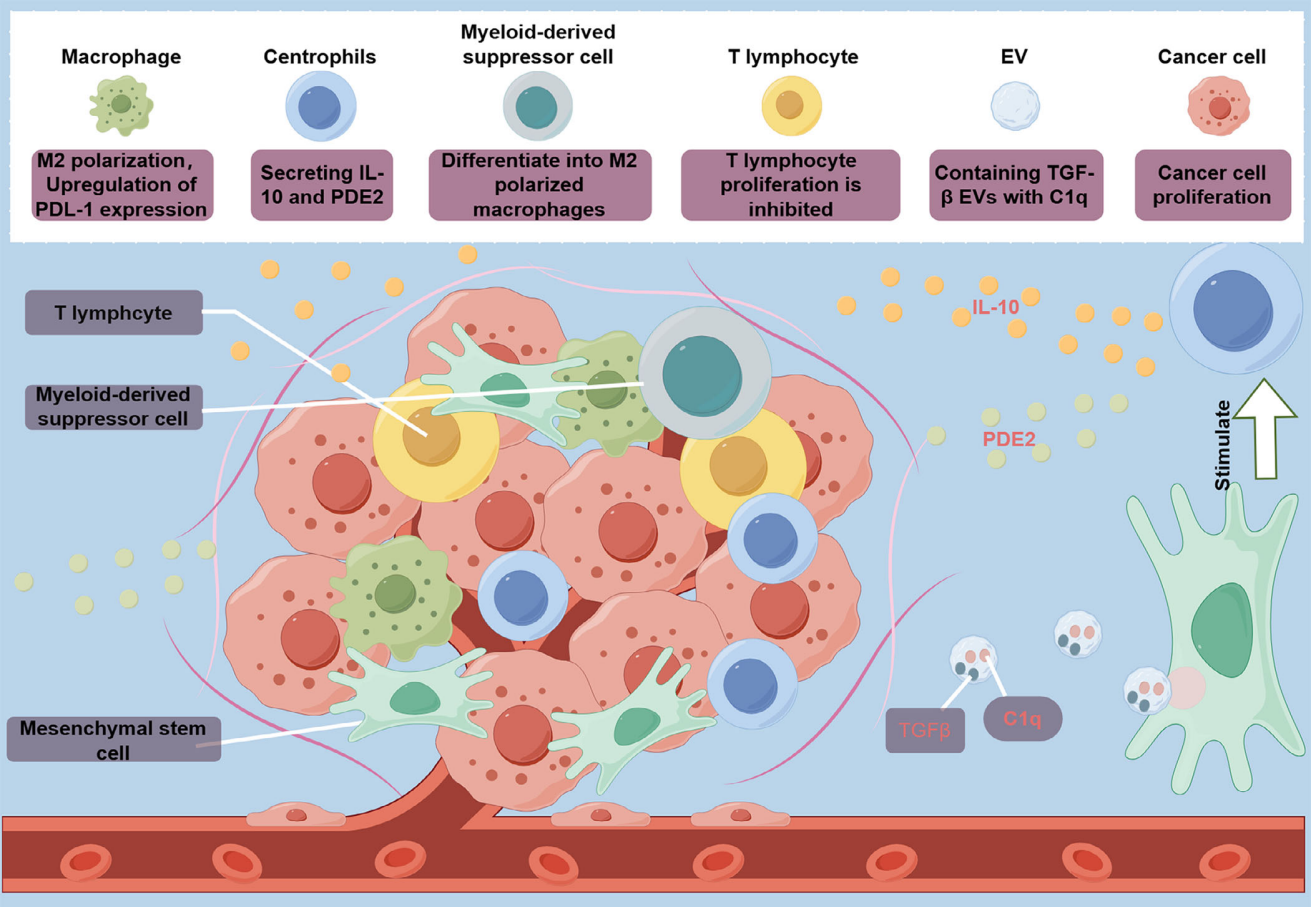
Immunosuppressive effect of MSCs in the development of malignant tumor. The differentiation of bone marrow‐derived suppressor cells into highly immunosuppressive M2‐type macrophages with upregulated expression of PDL‐1 can be facilitated by MSCs‐EVs through TGFβ and C1q. Furthermore, mesenchymal stem cells stimulate the secretion of IL‐10 and PGE2 by central granulocytes, resulting in the inhibition of T lymphocyte proliferation in the TME.

The majority of malignant cells can be identified, monitored, and ultimately eliminated by the immune system. However, a small portion of malignant cells are able to avoid detection and enter a phase known as “equilibrium,” leading to immune escape.^130^, ^131^, ^132^, ^133^, ^134^ These mechanisms involve modifications to both the tumor cells and the TME.^135^ Similarly, the suppression of immune function in the TME means that tumor cells are more likely to undergo immune escape and survive (Figure 2). Moreover, MSCs possess strong immunosuppressive properties that assist tumor cells in evading immune surveillance.^136^, ^137^, ^138^

The immunosuppressive capacities of MSCs within TMEs are significantly shaped by the immunomodulatory molecules they secrete and their capacity to induce the generation of immune‐suppressive cell populations. Notably, MSCs possess the ability to modulate immune homeostasis by directly interacting with various innate and adaptive immune cell types, including natural killer (NK) cells, T lymphocytes, and macrophages,^18^, ^139^ thereby dampening the cascade of immune responses and fostering tumor immune evasion. The body's immune system can eliminate cells undergoing malignant transformation through mechanisms involving NK cells and restricted cytotoxic T lymphocytes, whose functions are regulated by regulatory T cells (Tregs).^140^, ^141^, ^142^, ^143^, ^144^, ^145^ MSCs can promote the proliferation of Treg cells and inhibit mitogen‐induced T cell proliferation via cell cycle protein D2, leading to cell cycle arrest in the G0/G1 phase.^146^, ^147^ MSCs' immunosuppressive behavior is reportedly induced by their secretion of immunomodulatory molecules such as IL‐1α, IL‐4, IL‐6, TGFβ, and INF‐γ, particularly within the TME.^148^ Chang et al.^149^ demonstrated that coinjection of MSCs and the mouse prostate cancer cell line RM‐1 into mice resulted in upregulation of TGF‐β expression in MSCs due to IL‐1α secretion by RM‐1 cells, thereby promoting tumor development. The secretion of TGFβ1 by MSCs and the proliferation of Tregs, especially FoxP3‐expressing Tregs, are closely associated with immunosuppression.^149^, ^150^, ^151^ Additionally, MSCs stimulate tumor‐associated neutrophils to adopt an anti‐inflammatory phenotype and release IL‐10 and prostaglandin E2 (PGE2), suggesting their role in regulating neutrophil activity. In a breast tumor model, coculture of MSCs and neutrophils (CD11b+Ly6G+) demonstrated that neutrophils acquire immunosuppressive activity, inhibiting T cell proliferation in vitro and promoting tumor progression in vivo.^152^

MSCs can also regulate immune cells within the TME by secreting EVs containing immunomodulatory molecules. Reports have highlighted the crucial role of EVs, particularly those derived from MSCs, in mediating immune regulation between immune and nonimmune cells.^153^, ^154^ One study revealed that exosomes derived from MSCs (MSCs‐exos) obtained from both human and murine tumors enhance the malignant growth of breast cancer cells by inducing myeloid‐derived suppressor cells, which differentiate into highly immunosuppressive M2‐polarized macrophages within the TME. The underlying mechanisms involve the presence of TGF‐β, C1q, and semaphorins within MSCs‐exos, which contribute to myeloid tolerance by promoting the upregulation of PD‐L1 in immature myelomonocytic precursors and CD206+ macrophages. Additionally, these elements induce the differentiation of MHC class II+ macrophages, enhancing L‐Arginase activity and IL‐10 secretion at the tumor site, thereby promoting cancer progression.^155^

In summary, immune evasion within the TME, orchestrated by the modulation of immunomodulatory molecules secreted by MSCs, marks the initial imperative step for malignant tumor survival.

### 3.2 Angiogenesis promotion

After successfully evading the immune system, cancer cells require sufficient nutrients in order to survive. The proliferation and metastasis of tumor cells depend on a sufficient supply of oxygen and nutrients. The most effective way for them to acquire these nutrients is by forming new blood vessels around the tumor cells, thereby providing the necessary nourishment for the malignant tumor cells. Angiogenesis is the physiological process through which new blood vessels form from pre‐existing vessels, and this process serves to remove disposals and provide nutrients for cancer cells, thus playing an essential role in the occurrence and development of cancer.^156^, ^157^, ^158^

MSCs have been shown to stimulate angiogenesis, as evidenced by relevant reports highlighting the potential therapeutic applications of this characteristic in the treatment of severe ischemic limb diseases.^159^, ^160^, ^161^ Likewise, the property of MSCs to induce angiogenesis within tumor tissues can be exploited by tumor cells. High levels of cytokines and angiogenesis‐stimulating growth factors, such as IL‐6, IL‐8, βFGF, FGF‐2, PDGF, VEGF, TGFβ, and angiopoietin, are released by MSCs, thereby promoting tumor angiogenesis.^162^ It has been demonstrated in studies that the ability to induce angiogenesis is higher in breast cancer models when MCF‐7 cells are coinjected with EVs derived from bone marrow MSCs.^163^ And accumulated evidence has demonstrated that MSCs‐exos harbor angiogenic stimulatory factors, including TGFβ1, IL‐8, FGF, VEGF, which possess the ability to promote angiogenesis.^164^, ^165^

Notably, VEGF is recognized as a significant mediator of angiogenesis in cancer and can also exert its effects on tumor cells through paracrine signaling.^166^ Research conducted by Clavreul et al.^167^ has demonstrated that the coinjection of MSCs with glioma cells into nude mice resulted in an increase in angiogenesis. This augmentation was attributed to the secretion of various factors, including TGF‐β1, PDGF‐BB, hepatocyte growth factor (HGF), and SDF‐1/CXCL12, by these cells.^167^, ^168^ Furthermore, Orecchioni et al.^169^ found in a breast cancer model a dynamic interaction between endothelial cells and AD‐MSCs, leading to the formation of pericytes and mature vessels. This interconnection can be mediated via intercellular or paracrine signals, potentially involving angiopoietin signaling. Additionally, the expression of angiopoietin‐1 has been observed in AD‐MSCs, which plays a crucial role in promoting angiogenesis in breast cancer.^169^, ^170^

The promotion of malignant tumor angiogenesis by MSCs provides stable nutrition sources for cancer cells, laying an essential foundation for the progression and metastasis of cancer. Consequently, much of the current research is primarily focused on the inhibition of the proangiogenic activities of MSCs within the TME to enhance antiangiogenic therapy.^171^ For instance, Huang et al.^172^ found that IL‐6 secreted by MSCs can increase the secretion of endothelin‐1 (ET‐1) in human colorectal cancer cells, which induces the activation of Akt and ERK in endothelial cells, thereby enhancing their capacity for recruitment and angiogenesis to the tumor. Knocking out ET‐1 in colorectal cancer cells can inhibit ERK and Akt signaling in host endothelial cells, thereby weakening angiogenesis inhibition of tumor growth.^172^ Furthermore, Gao et al.^173^ also found that miR‐195a‐3p inhibits angiogenesis by targeting Mmp2 in murine MSCs, suggesting that miR‐195a‐3p may also play a role in regulating MSCs to inhibit angiogenesis and thus suppress malignant tumor development. It is evident that inhibiting the angiogenic capabilities of MSCs in the TME holds potential for suppressing tumor development.

### 3.3 Promote cancer cell proliferation

**FIGURE 3:**
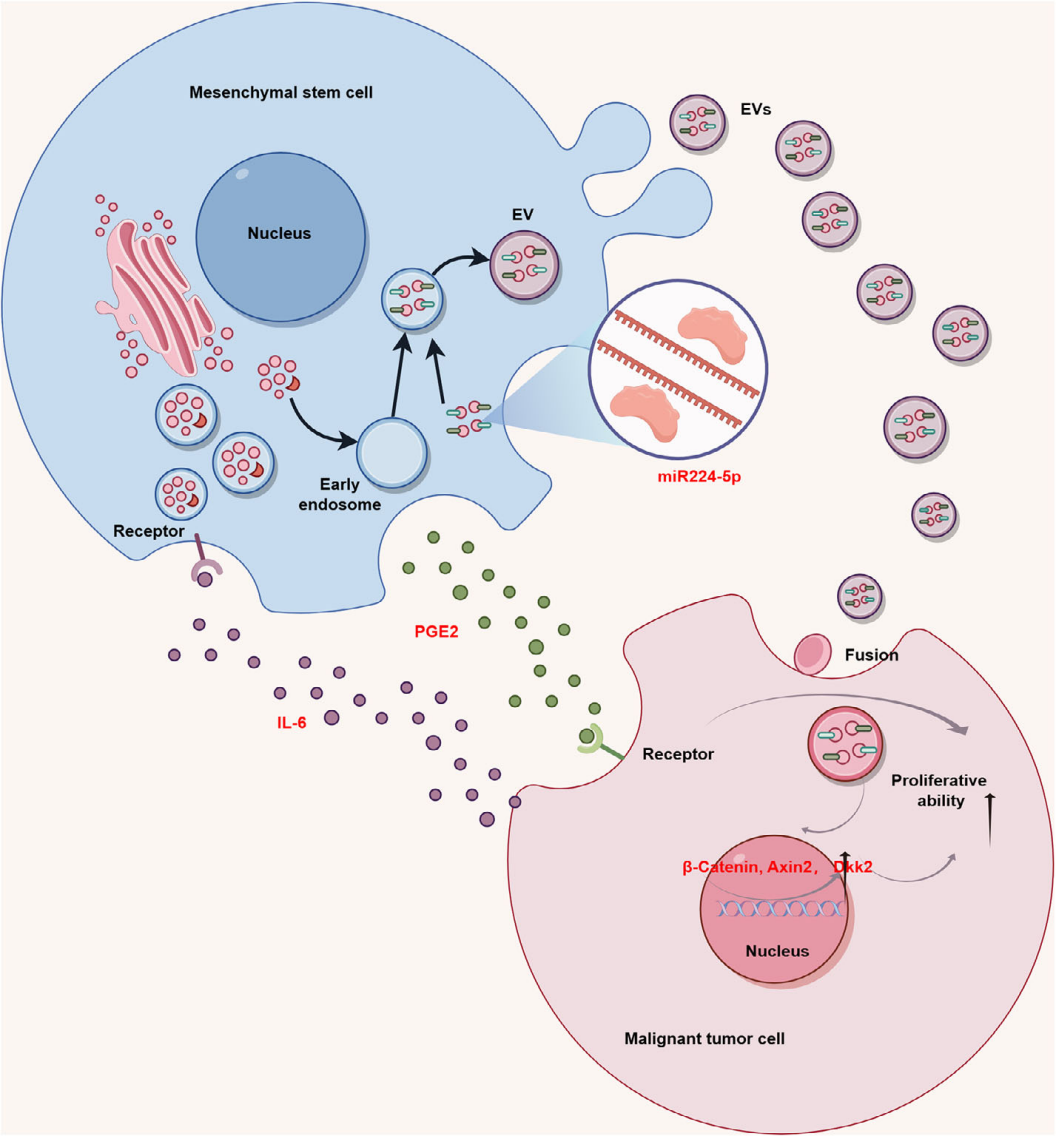
The role of MSCs in the proliferation of malignant tumor cells. malignant tumor cell proliferation can be induced by MSCs‐EVs through the delivery of miRNAs such as miR224‐5p and the promotion of gene expression, including Axin2 and Dkk1; MSCs can inhibit apoptosis in malignant tumor cells and induce autophagy, ultimately promoting malignant tumor cell proliferation, through the paracrine secretion of cytokines such as IL‐6 and PDGE2.

Cell proliferation is a complex process that primarily drives cell expansion. In the case of normal cells, proliferation remains in a dynamic equilibrium state. However, MSCs disrupt this balance in malignant tumor cells. Cancer cells, under the promotion of MSCs, undergo a series of complex interactions that result in immune evasion and angiogenesis. These interactions involve the release of EVs and paracrine signaling, ultimately leading to malignant tumor cell proliferation and disease progression (Figure 3). Studies have demonstrated the capability of MSCs to promote tumor growth in a mouse model of malignant tumors, and similar findings have been reported for MSCs coimplanted with cancer cells.^174^, ^175^

The role of extracellular vesicles (EVs) derived from MSCs (MSCs‐EVs) in cancer cell proliferation has been extensively explored and validated. Vallabhaneni et al.^163^ isolated MSCs‐derived EVs and characterized their cargo, revealing their ability to transport tumor‐regulating miRNA (miR), proteins, and metabolites, predominantly promoting malignant tumor cell proliferation.^176^, ^177^, ^178^, ^179^ For instance, miR‐224‐5p extracted from human umbilical cord‐derived MSCs‐exosomes was found to enhance breast cancer cell proliferation both in vivo and in vitro.^180^ Another study illustrated that exosomes derived from adipose‐derived MSCs (ADMSCs‐exos) promote MCF7 cell proliferation and elevate the expression levels of β‐catenin and Wnt target genes, such as Axin2 and Dkk1.^181^ These findings suggest that ADMSCs‐exos activate the Wnt signaling pathway, further augmenting breast cancer cell proliferation. Additionally, Zhu et al.^182^ discovered that miR‐301b‐3p within MSCs‐EVs enhances gastric cancer cell proliferation by downregulating the expression of thioredoxin interacting protein (TXNIP). Moreover, MSCs‐EVs were found to deliver noncoding RNA activated by DNA damage to osteosarcoma cells, promoting the proliferation and migration capabilities of osteosarcoma cells via modulation of the miR‐30c‐5p/KLF10 signaling axis.^177^

Furthermore, the TME can trigger a signaling cascade within MSCs, leading to the production of paracrine factors that promote malignant tumor development. For example, IL‐6 derived from breast cancer cells induces PGE2 production by infiltrating MSCs, subsequently promoting breast cell growth. This establishes a feedback loop with prognostic implications for patient survival in clinical settings.^183^ Additionally, paracrine factors secreted by BM‐MSCs or ADMSCs‐exos, particularly IL‐6, significantly enhance ER‐positive breast cancer cell proliferation, such as MCF7, T47D, or ZR‐75‐1, both in vitro and in vivo, contributing to an overall progrowth phenotype for stromal MSCs across various contexts.^184^, ^185^ Chen et al.^186^ discovered that MSCs upregulate the c‐Myc‐HK2 signaling pathway in gastric cancer cells by secreting HGF, thereby promoting gastric cancer cell proliferation and migration. Similarly, Liu et al.^187^ demonstrated that adipose‐derived MSCs induce EMT in ovarian cancer cells and activate the TGF‐β signaling pathway to enhance ovarian cancer growth and metastasis, an effect that can be reversed by the TGF‐β inhibitor SB431542. This suggests that targeting adipose‐derived MSCs with TGF‐β inhibitors has therapeutic potential for blocking ovarian cancer development. Furthermore, studies have shown that MSCs significantly promote the proliferation of malignant tumor cells such as glioblastoma, oral cancer, and tongue squamous cell carcinoma.^188^, ^189^, ^190^, ^191^, ^192^, ^193^

Through meticulously executed mechanistic studies and comprehensive validations utilizing both cellular and animal models, it has been unequivocally demonstrated that MSCs play a pivotal role in enhancing the proliferation of malignant tumor cells. This revelation underscores the critical part MSCs fulfill within the TME and their consequential potential as therapeutic targets.

### 3.4 Correlating MSCs with CSCs

The concept of CSCs suggests that tumors, akin to their normal tissue counterparts, possess a cellular hierarchy with CSCs positioned at the apex. CSCs are responsible for maintaining tumorigenicity and reproducing the inherent cellular heterogeneity within the primary tumor.^194^

Malignant tumor cells possess the capability to persist as CSCs within the bone marrow over extended periods, underscoring the clinical hurdles associated with their subsequent resurgence.^195^ Additionally, mounting evidence suggests that CSCs play a pivotal role in tumor metastasis and likely contribute to relapses following radiation therapy and chemotherapy.^196^ The investigation conducted by Sandiford et al.^197^ unveils a close correlation between the dormancy of breast CSCs (BCSCs) within the bone marrow and the activity of MSCs‐EVs. It has been observed that MSCs‐EVs instigate the onset of breast cell dormancy through a direct, stepwise dedifferentiation process, culminating in the generation of BCSCs. This transition occurs upon the interaction of breast cancer cells with MSCs within the perivascular niche of the bone marrow. The activation of the Wnt/β‐catenin pathway by MSCs‐EVs initiates a progressive dedifferentiation of breast cancer cells, ultimately leading to their conversion into quiescent BCSCs harbored within the bone marrow. Moreover, it has been discovered that breast cancer cell progenitors exhibit heightened responsiveness to MSCs‐EVs, prompting their dedifferentiation in vivo and the activation of multipotent signaling pathways reminiscent of those observed in BCSCs.^197^ Liu et al.^198^ have delineated the establishment of a cellular hierarchy by MSC and CSC populations, wherein BCSCs are modulated by MSCs expressing aldehyde dehydrogenase through cytokine loops involving CXCL7 and IL‐6. Upon interaction between IL‐6 secreted by BCSCs and IL‐6R/gp130 expressed on MSCs, MSCs upregulate CXCL7 expression. Consequently, the secretion of several cytokines, including IL‐8, IL‐6, CXCL5, and CXCL6, is induced by CXCL7 from both MSCs and BCSCs. It has been indicated that these cytokines promote the invasive properties and proliferation of BCSCs, with IL‐6 facilitating the homing of MSCs to tumor sites in mouse xenograft models.^71^, ^198^ Furthermore, in colorectal cancer, PGE‐2 is released by MSCs in response to IL‐1 secreted by tumor cells. This signaling cascade, operating in an autocrine manner, facilitates the upregulation of IL‐6, IL‐8, RANTES, CXCL1, and GRO‐α expression. Collectively, these factors contribute to the promotion of CSC formation.^199^

Therefore, a comprehensive exploration of the interplay between MSCs and cancer stem cells holds significant importance for the future development of targeted therapeutics.

### 3.5 Multilineage differentiation of MSCs in the TME

The differentiation capacity of MSCs as adult stem cells has been firmly established, and recent findings suggest that the TME can exert an impact on the differentiation potential of MSCs. It is widely recognized that cancer‐associated fibroblasts (CAFs) play a pivotal role in the TME, engaging in various functions such as matrix deposition, remodeling, reciprocal signaling interactions with cancer cells, and crosstalk with infiltrating leukocytes.^200^, ^201^, ^202^ The work by Kalluri^203^ has proposed that quiescent fibroblasts, including MSCs, have the ability to give rise to CAFs. Substantiating this hypothesis, in vitro studies have shown that prolonged exposure of MSCs to tumor cell‐conditioned medium can induce their transition into CAF‐like cells. Mishra et al.^204^ cultured human MSCs in breast cancer cell line (MDA‐MB‐231) conditioned medium for up to 30 days, resulting in increased expression of α‐smooth muscle actin (α‐SMA), fibroblast‐specific protein 1 (FSP‐1), stromal‐derived factor‐1α (SDF‐1α), and vimentin. These transformed MSCs also exhibited the ability to promote tumor cell growth in both in vitro and in vivo models.^204^ The mechanisms by which MSCs are educated in the TME to adopt CAF profiles are not fully understood, but there are noteworthy reported mechanisms worth considering. One such mechanism involves the influence of mechanical cues in the TME. MSCs cultured in a stiff ECM were found to upregulate expression of α‐SMA and release prosaposin, which subsequently enhanced cancer cell growth in vivo.^205^

**FIGURE 4:**
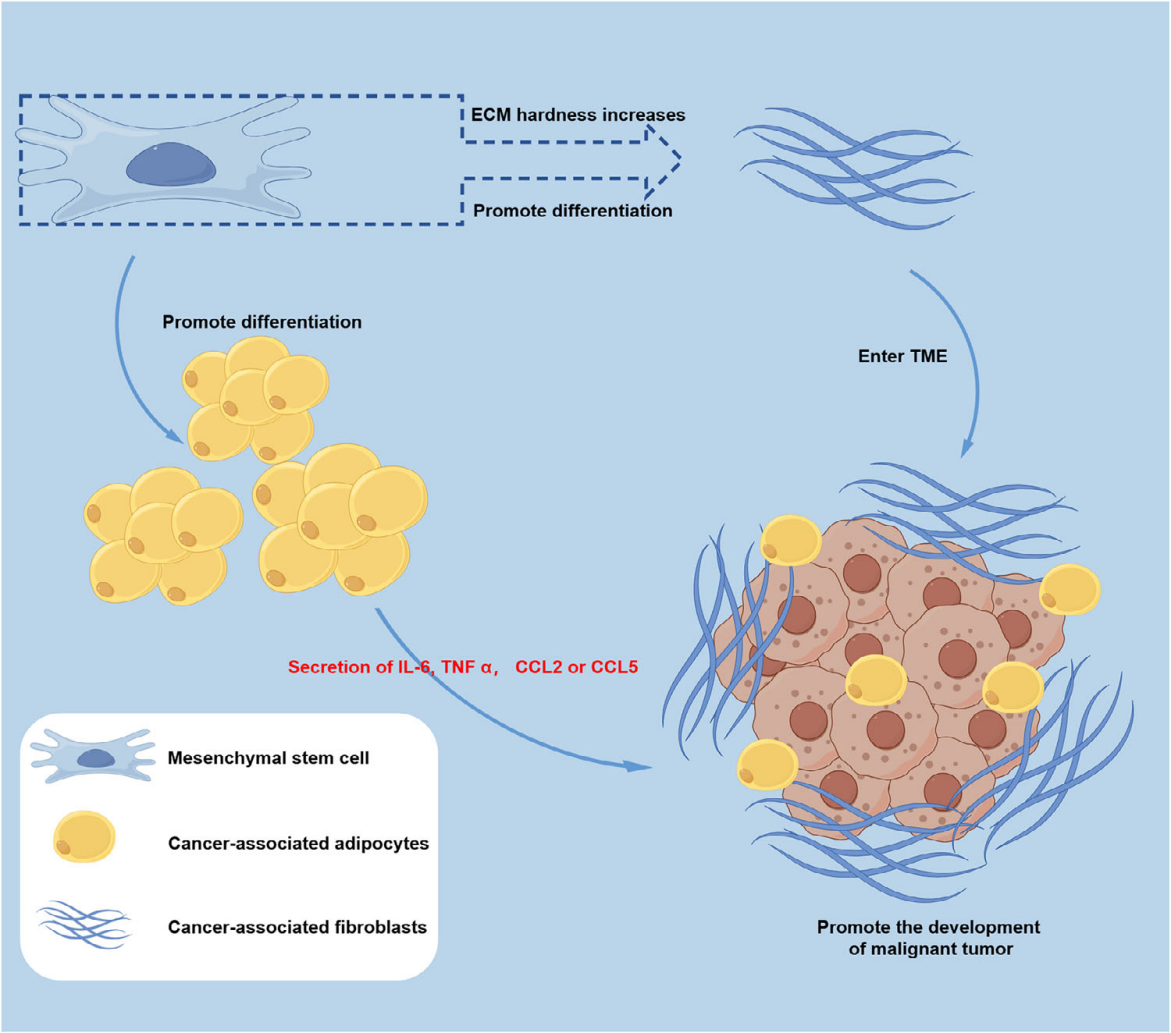
Differentiation of MSCs in TME. The differentiation of MSCs into CAFs can be facilitated by the TME, which is also correlated with the stiffness of the extracellular matrix; The TME promotes the differentiation of MSCs into CAAs and induces the secretion of IL‐6, TNF‐α, CCL2, and CCL5, thereby promoting the progression of malignant tumor.

In addition, MSCs have the capability to differentiate into adipocytes, which are implicated in the formation and function of cancer‐associated adipocytes (CAAs) within the TME.^206^, ^207^ These CAAs modulate the behavior of cancer cells by releasing proinflammatory mediators such as IL‐6, TNF‐α, CCL2, or CCL5, thereby promoting angiogenesis, proliferation, and metastasis.^208^, ^209^, ^210^ Figure 4 elucidates the differentiation of MSCs into CAFs and CAAs within the TME, ultimately contributing to the further deterioration of malignant tumor. This finding highlights the importance of investigating the regulatory mechanisms of MSC differentiation within the TME in future research endeavors.

### 3.6 The role of MSCs in promoting treatment resistance in cancer

The treatment of malignant tumors encompasses a variety of strategies, including surgery, chemotherapy, radiotherapy, targeted therapies, and more recently, immunotherapies.^211^, ^212^, ^213^, ^214^ Among them, chemotherapy is one of the main treatment approaches for cancer that has spread from the primary tumor site, and chemotherapy resistance is an important hindrance to achieving therapies in patients and is the crucial cause of death in most progressive stage cancers.^215^, ^216^ Drug resistance is caused by multiple factors, including genetic mutations and/or epigenetic changes as well as other cellular and molecular mechanisms.^217^ Previous studies have shown that MSCs contribute to treatment resistance through various pathways.^218^

Recent studies have demonstrated that MSCs‐EVs can induce resistance of cancer cells to chemotherapy drugs. Zhu et al.^182^ observed that MSCs‐EVs can induce resistance of gastric cancer cells to cisplatin/vincristine, a phenomenon associated with the upregulation of miR‐301b‐3p and downregulation of TXNIP. Additionally, other research indicates that MSCs‐exos released after doxorubicin treatment are more effective than BM‐MSCs‐exos in inducing breast cancer cell resistance to doxorubicin. This is primarily attributed to doxorubicin inducing the expression of miR‐21‐5p in MSCs and MSCs‐exos, consequently upregulating the expression of S100A6 in breast cancer cells, thus enhancing cell viability and Dox resistance.^219^ Conversely, Jia et al.^220^ found that miR‐1236 transferred by adipose‐derived MSCs‐exos increases breast cancer cell sensitivity to cisplatin by downregulating SLC9A1 and deactivating Wnt/β‐catenin. This demonstrates that MSCs‐exos exhibit a dual role in cancer chemotherapy, highlighting the need for further elucidation of the specific mechanisms involved.^221^ Furthermore, Skolekova et al.^221^ found that the secretory phenotype and behavior of MSCs exposed to cisplatin differed from those of naïve MSCs. The MSCs exhibited increased resistance to the cytotoxic effects of cisplatin, which typically induces apoptosis in tumor cells. On the contrary, a subset of the MSC population underwent senescence. However, pretreatment of MSCs with cisplatin resulted in alterations in the phosphorylation profiles of numerous kinases as well as an upregulation in the secretion of cytokines such as IL‐6 and IL‐8. These changes in cytokine and phosphorylation profiles ultimately led to enhanced chemoresistance and stemness in breast cancer cells.

These studies effectively demonstrated the impact of MSCs on malignant tumors chemotherapy agents. However, given the ongoing debate surrounding the role of MSCs in malignant tumor cell drug resistance, future investigations could delve deeper into their specific mechanisms of action, thereby providing a stronger theoretical foundation for the development of relevant drugs.

## 4 POTENTIAL ROLES OF MSCS IN CANCER TREATMENT

**FIGURE 5:**
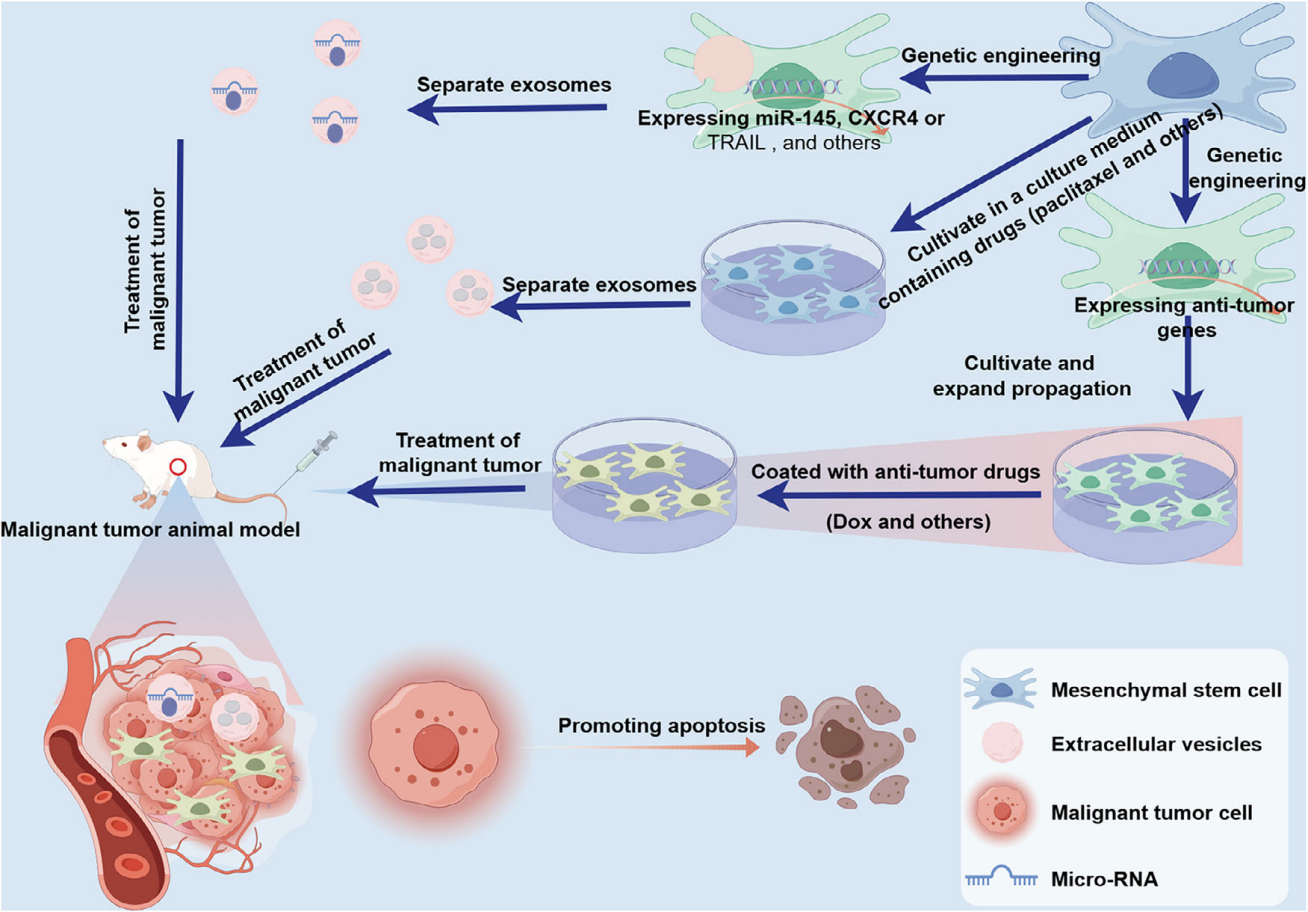
The potential of MSCs in the treatment of malignant tumor. Malignant tumors are being targeted for treatment through the use of genetically engineered MSCs that express antitumor genes and deliver chemotherapeutic agents such as doxorubicin (Dox) and others. Additionally, MSCs‐exos facilitate the transfer of miRNAs, including miR‐199a and others, which inhibit the development of malignant tumor cells. Furthermore, MSC‐exos loaded with paclitaxel selectively target malignant tumors at lower drug concentrations, effectively inhibiting their growth and metastasis.

Presently, treatment modalities for solid malignant tumors encompass surgical intervention, chemotherapy, radiation therapy, and immunotherapy.^222^, ^223^, ^224^ Surgical treatment is effective for early‐stage cancer without metastasis, but postoperative recurrence is common.^225^, ^226^ Chemotherapy and radiotherapy are adjuvant treatments for surgical treatment, but they often face drug resistance and toxic side effects due to their inability to differentiate between rapidly dividing normal cells and cancer cells.^227^, ^228^ Despite significant advancements in enhancing the efficacy of immunotherapy, therapies like anti‐PD‐1/PD‐L1 demonstrate limited antitumor effects. For instance, only patients with specific cancer subtypes, such as non‐small‐cell lung cancer (NSCLC), can benefit from anti‐PD‐1/PD‐L1 immunotherapy.^229^ Obviously, these current treatment methods are facing challenges. Therefore, in order to reduce or eliminate recurrence, drug resistance, and toxic side effects while ensuring a good quality of life for malignant tumors patients, more attention should be paid to the research of new therapies for malignant tumors. In the following section, we will discuss the potential role of MSCs in malignant tumors treatment (Figure 5).

### 4.1 MSCs as a therapeutic vector

In the past two decades, increasing interest has been observed in the potential of MSCs to migrate to tumor sites. The remarkable tumor tropism exhibited by MSCs provides an intriguing opportunity for the targeted delivery of antitumor agents.^230^ Recently, MSCs have been widely utilized for the delivery of therapeutic agents.^231^, ^232^, ^233^ Numerous studies have demonstrated the superior efficiency of MSCs as a cell‐mediated drug delivery system. Extensive research is underway exploring the use of MSCs for delivering gene therapeutic agents, chemotherapeutics, and oncolytic viruses (OVs) to achieve precise treatment of malignant tumors, yielding promising outcomes.^234^, ^235^, ^236^, ^237^, ^238^, ^239^, ^240^, ^241^, ^242^, ^243^ Table 1 summarizes additional research details in recent years utilizing MSCs as therapeutic carriers to target tumor cells and inhibit tumor development.

**TABLE 1:** Mesenchymal stem cells as carriers for loading antitumor agents.

MSCs sources	Cargo	Loading approaches	Tumor types	References
MB‐MSCs	TRAIL protein	Highly efficient adenoviral serotype 35 vectors were utilized to overexpress the TRAIL protein in MB‐MSCs.	Glioma	^234^ ^]^
MB‐MSCs	Oncolytic adenovirus	To infect MB‐MSCs directly, oncolytic adenovirus was utilized.	Lung adenocarcinoma and epidermoid carcinoma	^235^
BM‐MSCs	Theranostic sodium iodide symporter (NIS)	BM‐MSCs were genetically modified to express the NIS gene under the regulation of the cytomegalovirus (CMV) promoter.	Hepatocellular cancer	^236^
BM‐MSCs	TRAIL protein	Plasmids carrying the sequence of the TRAIL gene were transfected into BM‐MSCs.	Melanoma	^237^
BM‐MSCs	miR101	The miR101‐loaded MSCs were assembled using the layer‐by‐layer technique with gelatin and alginate miR101.	Hepatocellular cancer	^238^
AD‐MSCs	Oncolytic adenovirus	To infect AD‐MSCs directly, oncolytic adenovirus was utilized.	Lung carcinoma	^240^
BM‐MSCs	Apoptin	A recombinant adenovirus expressing Apoptin was constructed and used to infect the BM‐MSCs.	Hepatocellular cancer	^239^
UC‐MSCs	Doxorubicin	Doxorubicin was coincubated with UC‐MSCs in PBS.	Breast cancer	^241^
BM‐MSCs	HSV‐TK	Using lentiviral vectors, the HSV‐TK gene was introduced into BM‐MSCs.	Glioblastoma	^242^
AD‐MSCs	Curcumin	Curcumin‐loaded nanoparticles were prepared using biotinylated chitosan polymer and anchored onto AD‐MSCs via biotin‐avidin binding.	Melanoma	^243^

Abbreviations: MB‐MSCs, menstrual blood‐derived mesenchymal stem cells; UC‐MSCs, umbilical cord‐derived mesenchymal stem cells.

In 2002, the use of bioactive molecules in therapy took a significant step forward with the overexpression of IFN‐β by engineered MSCs.^244^ Subsequently, MSCs have demonstrated their ability to deliver a diverse range of cytokines and growth factors identified as antitumor agents to the tumor site, including IL‐2, IL‐7, IL‐12, IL‐15, IL‐18, IL‐25, IFN‐α, IFN‐γ, and NK4.^245^ Furthermore, Conrad and colleagues^246^ undertook the engineering of murine MSCs to express reporter genes or therapeutic genes under the selective control of the Tie2 promoter/enhancer. Following injection into the peripheral circulation of mice with either orthotopic pancreatic or spontaneous breast cancer, the engineered MSCs exhibited active recruitment to the growing tumor vasculature and induced selective expression of either reporter red fluorescent protein or suicide genes [herpes simplex virus‐thymidine kinase (HSV‐TK) gene] when the adoptively transferred MSCs developed endothelial‐like characteristics. The HSV‐TK gene product, in combination with the prodrug ganciclovir (GCV), generates a potent toxin that affects replicative cells. The homing of engineered MSCs, along with the selective induction of HSV‐TK in concert with GCV, resulted in the creation of a toxic tumor‐specific environment. The efficacy of this approach was demonstrated by a significant reduction in primary tumor growth and an extension of overall survival in both tumor models.^246^ It has also been noted by researchers that the introduction of the HSV‐TK gene into MSCs using lentiviral vectors is effective in interfering with the development of glioblastoma.^242^ Additionally, the incorporation of TRAIL protein into MSCs has shown the ability to inhibit the progression of glioma and melanoma.^234^, ^237^ In summary, the aforementioned studies have provided evidence to illustrate the capacity of MSCs to function as carriers for gene therapy agents in the battle against tumors.

Furthermore, it is well recognized that while chemotherapy continues to be an effective and primary therapeutic approach for malignant tumors, its effectiveness is impeded by the significant systemic toxicity it induces.^247^ The lack of precise tumor cell targeting by chemotherapeutic agents inevitably results in damage to normal cells, which is the primary cause of systemic toxic reactions associated with chemotherapy. Recent studies have demonstrated that the delivery of anticancer drugs via MSCs can significantly improve the tumor‐targeting specificity of the drugs.^248^ For example, Cao et al.^241^ utilized human umbilical cord‐derived MSCs (hUC‐MSCs) loaded with transferrin‐inspired nanoparticles containing doxorubicin (Tf‐inspired‐NPs) to enhance the efficacy of antitumor treatment. The findings demonstrated that hUC‐MSCs loaded with Tf‐inspired‐NPs not only demonstrated the controlled‐release capability of Tf‐inspired‐NPs but also incorporated the tumor‐targeting and penetrating abilities inherent to MSCs. Nude mice with breast cancer (MCF‐7) treated with hUC‐MSCs carrying Tf‐inspired‐NPs exhibited a significant reduction in tumor volume compared with those treated with free doxorubicin or Tf‐inspired‐NPs alone.^241^ In the study by Xu et al.,^243^ a cell–nanoparticle hybrid vector was constructed using MSCs as the targeting cellular carrier and biotinylated chitosan polymer nanoparticles as the drug depot. Drug‐loaded nanoparticles were prepared by encapsulating the hydrophobic model drug curcumin into biotinylated chitosan polymer. The biotin‐modified nanoparticles were anchored on the surface of biotinylated MSCs through biotin‐avidin binding. Both in vitro and in vivo antitumor results support the potential benefits of the cell–nanoparticle hybrid vector in the therapy of pulmonary melanoma metastasis.^243^ These studies collectively highlight the advantages of using MSCs for targeted delivery of anticancer drugs.

OVs are viruses, whether natural or recombinant, that possess the capability to eradicate cancer cells.^249^ However, systemic administration of oncolytic adenoviruses is often hindered by poor tumor targeting, limiting the effectiveness of virotherapy for disseminated cancers. To address this issue, the use of MSCs as cellular carriers for OVs has been explored.^250^ The study conducted by Barlabé et al.^235^ demonstrated that loading OVs onto MSCs enhances their antitumor effects and further improves their targeting capabilities by augmenting the expression of genes associated with the epidermal growth factor receptor. Although there are limited studies on the use of MSCs for OV delivery, this approach holds great potential for precise targeted therapy against malignant tumors.

While current research has highlighted the potential of MSCs as carriers for targeted delivery to tumor tissues, two significant challenges must be addressed before the translation of MSC‐based cancer therapies into clinical settings. On one hand, the dual roles of MSCs in cancer development, as both protumorigenic agents, have been extensively discussed in the preceding sections. Consequently, the debate continues regarding whether MSCs should be viewed as anticancer agents or as therapeutic targets in cancer treatment. Addressing how to mitigate the extrinsic tumorigenicity of exogenous MSCs, such as through functional modifications, will be a crucial area of focus. On the other hand, the distinct tropism abilities of different types of MSCs necessitate further elucidation of their homing patterns in various cancers. This delineation will provide valuable insights for selecting the appropriate MSCs for cancer therapy.

### 4.2 MSCs‐EVs as drug carriers for cancer therapy

EVs possess various biophysical properties, including biocompatibility, low toxicity, low immunogenicity, enhanced circulation stability, and bio‐barrier permeability. As a result, they can serve as effective carriers for drug delivery to enhance the regulation of target tissues and organs.^251^, ^252^, ^253^ Specifically, MSCs‐EVs can transport different types of cargo, such as natural products and chemotherapeutic agents, and selectively target specific cells for the treatment of malignant tumors.^41^, ^102^, ^180^, ^220^, ^254^, ^255^, ^256^, ^257^, ^258^, ^259^

Similar to MSCs, MSCs‐EVs also exhibit tumor tropism.^260^ As shown in Table 2, extensive research has been conducted to explore the use of MSCs‐EVs as vehicles for delivering antitumor substances, leading to significant breakthroughs.^261^, ^262^, ^263^, ^264^, ^265^, ^266^, ^267^, ^268^, ^269^, ^270^, ^271^, ^272^, ^273^, ^274^, ^275^ These studies mainly fall into two categories. One category involves delivering substances that can interfere with tumor gene expression, such as micro‐RNAs and Si‐RNAs, through MSCs‐EVs. For instance, Lou et al.^267^ infected MSCs with a lentivirus carrying miR‐199a, followed by isolation of exosomes. They then intravenously injected the exosomes containing miR‐199a into a liver cancer animal model along with doxorubicin for combination therapy. The results demonstrated that MSCs‐Exo containing miR‐199a could selectively distribute to tumor tissues and significantly enhance the in vivo antitumor effect of doxorubicin against liver cancer.^267^ The other category involves delivering antitumor drugs like paclitaxel and doxorubicin through MSCs‐EVs. For example, Wang et al.^268^ coincubated paclitaxel with MSCs‐Exo and then delivered the paclitaxel‐loaded MSCs‐Exo into a mouse colon cancer model. The results showed that this type of exosome significantly inhibited tumor growth in the CT26 tumor‐bearing mouse model.^268^

**TABLE 2:** Mesenchymal stem cell‐derived extracellular vesicles as carriers for loading antitumor agents.

MSCs‐EVs sources	Cargo	Loading approaches	Tumor types	References
UCB‐MSCs	miR‐503‐3p	The plasmid carrying the miR‐503‐3p sequence was transfected into UCB‐MSCs.	Endometrial cancer	^261^ ^]^
Not mentioned	CXCR4 protein and Si‐Survivin	First, MSCs were infected with lentivirus to obtain exosomes overexpressing CXCR4, following which Si‐Survivin was loaded via electroporation.	Lung and gastric cancer	^262^
BM‐MSCs	miR‐let‐7c	Transfect miR‐let‐7c directly into BM‐MSCs.	Prostate cancer	^263^
BM‐MSCs	Norcantharidin (NCTD)	NCTD was loaded into purified exosomes from BM‐MSCs via electroporation.	Hepatocellular carcinoma	^264^
AD‐MSCs	miR‑218	miR‑218 was loaded into purified exosomes from AD‐MSCs via electroporation.	Breast cancer	^265^
BM‐MSCs	TRAIL protein	The plasmid carrying the TRAIL sequence was transfected into BM‐MSCs.	Melanoma	^266^
AD‐MSCs	miR‐199a	MSCs were infected with lentivirus to obtain exosomes overexpressing miR‐199a.	Hepatocellular carcinoma	^267^
AD‐MSCs	Paclitaxel	Coincubating paclitaxel with exosomes from AD‐MSCs.	Colon carcinoma	^268^
UC‐MSCs	miR‐3182	miR‐3182 was loaded into purified exosomes from UC‐MSCs via electroporation.	Breast cancer	^269^
BM‐MSCs	5‑fluorouracil (5‑Fu)	5‐Fu was loaded into exosomes derived from BM‐MSCs through a process involving incubation and sonication.	Cholangiocarcinoma	^270^
DPSCs	miR‐34a	The plasmid carrying the miR‐503‐3p sequence was transfected into DPSCs.	Breast cancer	^271^
AD‐MSCs	miR‐145	AD‐MSCs were infected with lentivirus to obtain exosomes overexpressing miR‐145.	Breast cancer	^272^
BM‐MSCs	Doxorubicin	Coincubating doxorubicin with exosomes from BM‐MSCs.	Osteosarcoma	^273^
BM‐MSCs	miR‐205−5p	The plasmid carrying the miR‐205−5p sequence was transfected into BM‐MSCs.	Hepatocellular carcinoma	^274^
BM‐MSCs	Paclitaxel and gemcitabine monophosphate	Gemcitabine monophosphate was loaded into purified exosomes from BM‐MSCs via electroporation, and paclitaxel was loaded into exosomes from BM‐MSCs through a process involving incubation and sonication.	Pancreatic ductal adenocarcinoma	^275^

Abbreviations: DPSCs, dental pulp stem cells; UCB‐MSCs, umbilical cord blood‐derived mesenchymal stem cells.

While significant progress has been made in utilizing MSCs‐EVs for delivering antitumor substances, it is important to note that these advancements are still limited to the preclinical stage, and there is still a long way to go before clinical translation. Similar to MSCs, MSCs‐EVs also have a protumor effect. However, compared with MSCs, MSCs‐EVs are safer due to their lack of proliferative capability in vivo. Therefore, MSCs‐EVs still hold tremendous potential in tumor therapy.

### 4.3 The in vivo distribution and clearance of MSCs and MSCs‐EVs

The distinctive characteristic of cell‐based therapies, as opposed to traditional small molecule or biologic treatments, lies in their “living” nature. Due to the significant heterogeneity of MSCs, their fate within the body, including distribution and duration of persistence, constitutes crucial factors influencing both their efficacy and safety.^276^ The administration of MSCs can occur via systemic or topical routes. In the context of utilizing MSCs as therapeutic carriers for malignant tumors, systemic administration is typically favored. Intravenous administration, in particular, stands out for its simplicity, minimal invasiveness, and strong potential for clinical translation.^277^ However, intravenous administration is prone to nonspecific trapping in other tissues, especially the lungs, a phenomenon observed since the inception of MSC research.^278^ Intravenously administered, MSCs, typically exhibiting an average size range of 14−20 µm when in suspension, exceed the diameter of pulmonary capillaries (8–15 µm). Consequently, they are initially entrapped within the pulmonary vasculature before undergoing subsequent migration to organs such as the liver and spleen. Ultimately, they are cleared from the body.^279^, ^280^, ^281^ Under pathological conditions, the distribution of MSCs may deviate from the norm, as evidenced by their homing toward sites rich in malignant tumor tissue due to the secretion of chemoattractants and cytokines by tumor cells.^282^ The clearance of MSCs from the body may involve interactions with immune cells; studies in immunocompromised mice have shown MSC presence in multiple tissues for up to 120 days following intraperitoneal injection, whereas in immunocompetent counterparts, MSC survival is limited to around 20 days.^283^, ^284^ Reports suggest that MSCs emit “eat me” signals upon entry into the body, leading to their phagocytosis by engulfing cells, a potentially crucial clearance pathway.^285^ This phenomenon correlates with the migration of macrophages, NK cells (NK), and dendritic cells to sites of tissue injury.^286^ Thus, the use of MSCs as carriers for tumor therapy can be considered relatively safe. However, future research should delve deeper into the mechanisms underlying MSC clearance in vivo to ensure the safety of MSC‐based tumor therapies.

Intravenously administered, MSCs‐EVs exhibit a distribution pattern akin to that of MSCs, with predominant accumulation observed in the liver and spleen following infusion, before dispersing into other tissue organs.^287^ Notably, while MSCs display marked pulmonary retention, such retention is less pronounced with MSCs‐EVs. Bioluminescence and fluorescence‐mediated tomography in murine models reveal the primary sites of MSCs‐EVs localization to be the liver, kidneys, and spleen post intravenous injection. EVs are detectable in the brain, heart, and muscles within 30 min postinjection, and in urine after 60 min; however, detection of administered EVs within the body becomes markedly challenging after 6 h.^287^, ^288^ Furthermore, owing to the inability of MSCs‐EVs to self‐replicate, administration of MSCs‐EVs is generally considered safe.

## 5 CONCLUSIONS AND PERSPECTIVES

Over the past several decades, a wealth of evidence has steadily accumulated, highlighting the significant advancements made in our understanding of MSCs’ roles within the TME and their impact on tumor progression. While opinions diverge regarding the influence of MSCs on malignant tumors—with some studies suggesting a substantial promotive effect and others indicating the contrary—this discrepancy may stem from differences in the sources of MSCs used in research or the specific components of MSCs being examined for their effects on tumors.^289^, ^290^, ^291^ Nonetheless, it is evident that a greater number of reports support the notion that MSCs facilitate the development of various malignancies. Moreover, extensive research has revealed that MSCs contribute to a complex array of interactions within the TME, leading to tumor cell survival, proliferation, migration, and ultimately, metastasis, and deterioration.^292^ The mechanisms by which MSCs exert their influence in the TME include inducing immunosuppression, promoting angiogenesis, enhancing tumor cell proliferation, interacting with CSCs, enabling multilineage differentiation, and contributing to therapeutic resistance. Among these, immunosuppression and angiogenesis enable tumor cell survival within the TME, while proliferation, interaction with CSCs, and multilineage differentiation further exacerbate the tumor, with therapeutic resistance culminating in the termination of patient life. Thus, the process by which MSCs promote tumor progression is exceedingly intricate, and dissecting their roles and underlying mechanisms can provide deeper insights into the patterns of malignant tumor development, paving the way for the discovery of effective anticancer therapies.

Additionally, it is known that MSCs are distributed in various tissues throughout the body. The question arises as to how MSCs enter the TME and contribute to tumor exacerbation. A substantial body of research has gradually unveiled this mystery, revealing that tumors can induce MSCs to migrate toward them, similar to how wounds attract these cells.^293^ This tumor tropism of MSCs positions them as promising carriers for delivering antitumor agents, a potential that has been validated in numerous studies.^294^ Given that MSCs are living cells, their administration poses certain risks. Consequently, researchers have focused on utilizing MSCs‐EVs as vehicles for antitumor agent delivery, as MSCs‐EVs also exhibit homing capabilities to malignant tumors.^295^ Similarly, using MSCs‐EVs as carriers has been shown in many studies to effectively transport antitumor agents to the tumor site, achieving superior antitumor effects compared with the agents alone.^296^ However, both MSCs and MSCs‐EVs share a common challenge: their inherent promotive effect on tumor progression.

In summary, MSCs play a pivotal role in the occurrence and development of malignant tumors. Simultaneously, MSCs and their secreted EVs demonstrate tremendous potential as carriers for delivering therapeutic agents in future cancer treatments. Currently, it is urgent to enhance our understanding of the complex interactions between MSCs, cancer cells, and components of the TME to propose highly specific therapeutic strategies.

## AUTHOR CONTRIBUTIONS

Jian Tang wrote the initial draft. Xiaogang Mao and Kun Meng wrote the final version. Yu Chen, Mengjun Tang, and Hui Xing supervised the final version. Chunhua Wang, Ying Xia, and Tingyu Yu participated in drawing pictures. Yang Yang, Lijuan Yin, and Liang Shen participated in the discussion and the preparation of the manuscript. All authors have read and approved the final manuscript.

## CONFLICT OF INTEREST STATEMENT

The authors declare no conflict of interest.

## ETHICS STATEMENT

Not applicable.

## Data Availability

Not applicable.
